# *catena*-Poly[[[diaqua­(1,10-phenan­thro­line)manganese]-μ-3-[3-(carboxyl­ato­meth­oxy)phen­yl]acrylato] monohydrate]

**DOI:** 10.1107/S1600536812023896

**Published:** 2012-06-16

**Authors:** Jun Ji, Yuan-Fa Yang, Yi-Hang Wen

**Affiliations:** a Zhejiang Key Laboratory for Reactive Chemistry on Solid Surfaces, Institute of Physical Chemistry, Zhejiang Normal University, Jinhua, Zhejiang 321004, People’s Republic of China

## Abstract

The title compound, [Mn(C_11_H_8_O_5_)(C_12_H_8_N_2_)(H_2_O)_2_]·H_2_O, was obtained under hydro­thermal conditions. The coordination environment of the Mn(II) atom is a distorted MnN_2_O_4_ octa­hedron defined by two N atoms from 1,10-phenanthroline, two water O atoms and two carboxyl­ate O atoms from two acrylate anions. The bis-monodentate coordination mode of the anion leads to the formation of chains propagating in [010]. Inter­molecular O—H⋯O hydrogen bonds link the chains into a two-dimensional network parallel to (100). In the voids of this arrangement, disordered lattice water mol­ecules are present.

## Related literature

For the study of metal-organic frameworks, see: Zhang *et al.* (2008 ▶); Zheng *et al.* (2010 ▶); Wang *et al.* (2006 ▶); Yi *et al.* (2005 ▶). For related structures, including 1,10-phenanthroline as a ligand, see: Chen *et al.* (2005 ▶); Ma *et al.* (2005 ▶); For the coord­ination modes of carb­oxy­meth­oxy acids, see: Novitchi *et al.* (2005 ▶). 
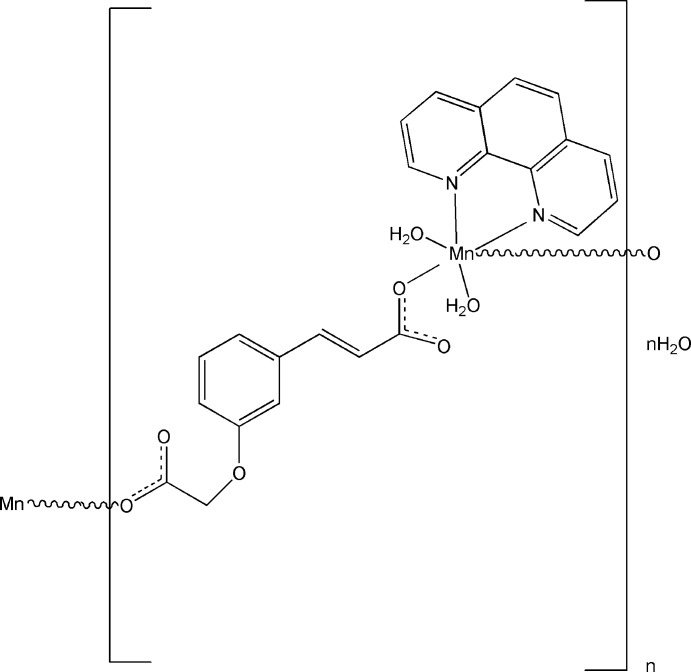


## Experimental

### 

#### Crystal data

[Mn(C_11_H_8_O_5_)(C_12_H_8_N_2_)(H_2_O)_2_]·H_2_O
*M* *_r_* = 509.37
Monoclinic, 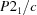

*a* = 12.8455 (4) Å
*b* = 21.5944 (7) Å
*c* = 8.2681 (3) Å
β = 93.712 (2)°
*V* = 2288.68 (13) Å^3^
*Z* = 4
Mo *K*α radiation
μ = 0.63 mm^−1^
*T* = 293 K
0.52 × 0.32 × 0.06 mm

#### Data collection

Bruker APEXII CCD diffractometer
Absorption correction: multi-scan (*SADABS*; Sheldrick, 1996 ▶) *T*_min_ = 0.79, *T*_max_ = 0.96
32833 measured reflections
4749 independent reflections
3716 reflections with *I* > 2σ(*I*)
*R*_int_ = 0.034

#### Refinement

*R*[*F* ^2^ > 2σ(*F* ^2^)] = 0.040
*wR*(*F* ^2^) = 0.116
*S* = 1.06
4749 reflections
314 parameters
6 restraints
H atoms treated by a mixture of independent and constrained refinement
Δρ_max_ = 0.49 e Å^−3^
Δρ_min_ = −0.36 e Å^−3^

### 

Data collection: *APEX2* (Bruker, 2006 ▶); cell refinement: *SAINT* (Bruker, 2006 ▶); data reduction: *SAINT*; program(s) used to solve structure: *SHELXS97* (Sheldrick, 2008 ▶); program(s) used to refine structure: *SHELXL97* (Sheldrick, 2008 ▶); molecular graphics: *DIAMOND* (Brandenburg, 1999 ▶); software used to prepare material for publication: *SHELXTL* (Sheldrick, 2008 ▶).

## Supplementary Material

Crystal structure: contains datablock(s) I, Mn. DOI: 10.1107/S1600536812023896/bq2356sup1.cif

Structure factors: contains datablock(s) I. DOI: 10.1107/S1600536812023896/bq2356Isup2.hkl

Additional supplementary materials:  crystallographic information; 3D view; checkCIF report

## Figures and Tables

**Table 1:** Hydrogen-bond geometry (Å, °)

*D*—H⋯*A*	*D*—H	H⋯*A*	*D*⋯*A*	*D*—H⋯*A*
O2*W*—H2*WB*⋯O3^i^	0.83 (2)	1.92 (2)	2.741 (2)	176 (3)
O2*W*—H2*WA*⋯O5^ii^	0.84 (2)	1.95 (2)	2.764 (2)	164 (3)
O1*W*—H1*WB*⋯O4^iii^	0.85 (2)	2.08 (2)	2.841 (3)	150 (3)
O1*W*—H1*WA*⋯O3^iv^	0.84 (2)	1.98 (2)	2.810 (2)	176 (3)

Symmetry codes: (i) 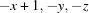; (ii) 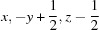; (iii) 
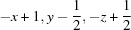
; (iv) 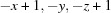.

**Fig. 1.**
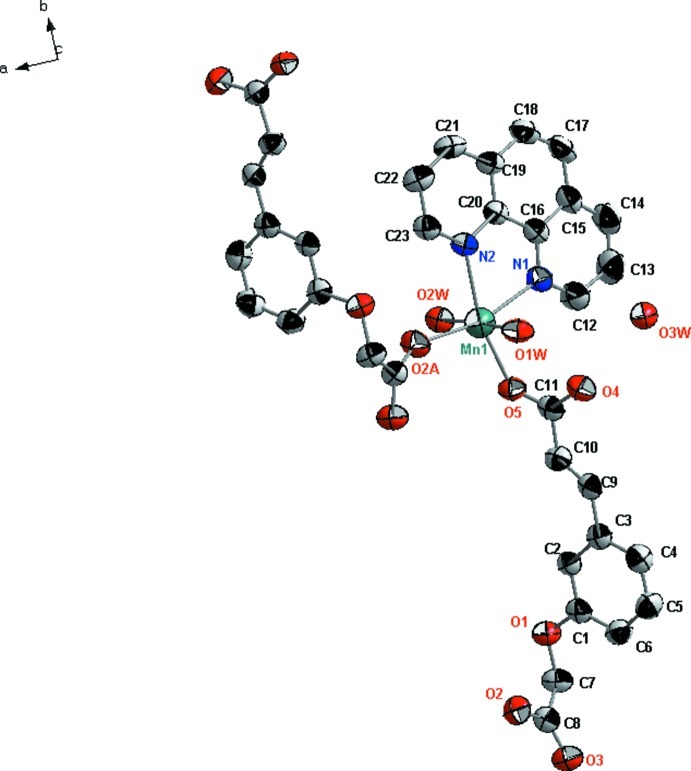
The molecular structure of title compound. Displacement ellipsoids are drawn at the 50% probability level. The disordered lattice water molecule is shown with an arbitrary radius. [Symmetry code:(A) -x + 1,y - 1/2,-z + 1/2.]

**Fig. 2.**
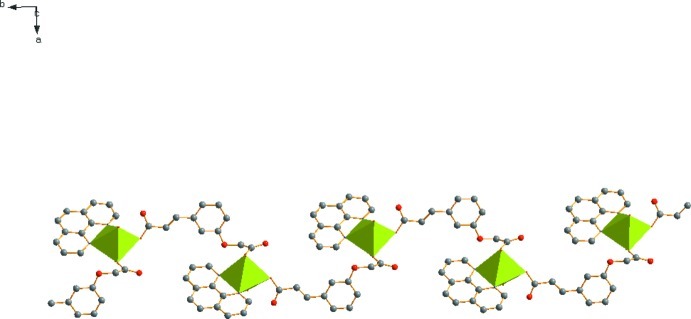
The chain structure of the title compound along the b axis.

**Fig. 3.**
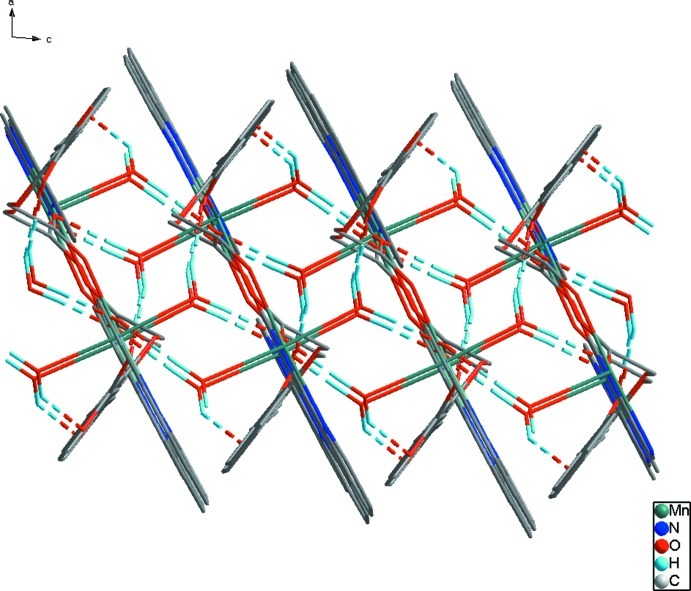
View of the supramolecular network connected by hydrogen-bonding interactions.

## supplementary crystallographic information

### Comment

Nowadays, the study of metal-orgnic fameworks (MOFs) has witnessed tremendous growth as one of the most crucial areas of material science (Zhang *et al.* (2008)). The rational design of these coordination polymers are of great interest in crystal engineering (Zheng *et al.*(2010)). Some valuable studied MOFs are based on carboxylate ligands because of their linking modes (Wang *et al.*(2006)). Using bridging ligands in assembly with metal cations of diverse geometries can induce complex structures and variable topologies (Yi *et al.*(2005)). Related information about carboxymethoxy acid and 1,10-phenanthroline can be obtained by these reports (Chen *et al.*(2005), Ma *et al.* (2005), Novitchi *et al.*(2005)). Herein we report the structure of the Mn title compound, [Mn(C_11_H_8_O_5_)(C_12_H_8_N_2_)(H_2_O)_2_]^.^H_2_O.

As shown in Figure 1, the asymmetric unit of the title complex is composed of one Mn(II) atom, one 3-carboxymethoxy phenyclacrylate anion, one 1,10-phenanthroline ligand, two coordinating water molecules and one solvent water molecule. The six-coordinate Mn(II) ions is surrounded in form of a distorted octahedron by two N atoms from 1,10-phenanthroline, two O atoms from L ligands and two O atoms from water molecules. In the title complex, 1,10-phenanthroline is a terminal ligand and L plays the role of a bis-monodentate bridging ligand. Each manganese site is connected by two µ_2_-L ligands, forming a zigzag chain along the *b* axis (Figure 2). There are four kinds of hydrogen-bonding interactions in the compound (Table 1), but only three link the chains to each other, making up a two-dimensional supramolecular network parellel to (100) (Figure 3).

### Experimental

A mixture of MnAc_2_^.^4H_2_O (0.2451 g, 1 mmol), 3-carboxymethoxy phenylacrylic acid (0.2220 g, 1 mmol) and 1,10-phenanthroline (0.0991 g, 0.5 mmol) was dissolved in 20 ml EtOH/H_2_O (*v*/*v*, 1:9). The pH value was then adjusted to 7 by 2 mol/*L* NaOH solution. The mixture was then sealed in a 25 ml stainless steel reactor and heated to 433 K for 3 days. Then the reactant mixture was cooled to room temperature at the rate of 5 K per hour. After evaporation of the resulting solution for a few days, yellow crystals of the title compound were obtained.

### Refinement

The carbon-bound H-atoms were positioned geometrically and included in the refinement using a riding model [C—H 0.93 Å *U*_iso_(H) = 1.2*U*_eq_(C)]. Water H atoms were located in different maps and refined with distance restraints of O—H = 0.85 (2) Å and H—H = 1.35 Å, with displacement parameters set at 1.5*U*_eq_(O). A solvent water molecule with a large displacement parameter is also present in the voids of the structure. It is disordered and has been refined only with an U_iso_ value and without its H atoms. The O···O contacts between the lattice water molecules are 2.525 Å.

### Crystal data

**Table:**

[Mn(C_11_H_8_O_5_)(C_12_H_8_N_2_)(H_2_O)_2_]·H_2_O	*F*(000) = 1052
*M**_r_* = 509.37	*D*_x_ = 1.478 Mg m^−^^3^
Monoclinic, *P*2_1_/*c*	Mo *K*α radiation, λ = 0.71073 Å
Hall symbol: -P 2ybc	Cell parameters from 9963 reflections
*a* = 12.8455 (4) Å	θ = 1.6–26.5°
*b* = 21.5944 (7) Å	µ = 0.63 mm^−^^1^
*c* = 8.2681 (3) Å	*T* = 293 K
β = 93.712 (2)°	Plate, yellow
*V* = 2288.68 (13) Å^3^	0.52 × 0.32 × 0.06 mm
*Z* = 4	

### Data collection

**Table:**

Bruker APEXII CCD diffractometer	4749 independent reflections
Radiation source: fine-focus sealed tube	3716 reflections with *I* > 2σ(*I*)
Graphite monochromator	*R*_int_ = 0.034
ω scans	θ_max_ = 26.5°, θ_min_ = 1.6°
Absorption correction: multi-scan (*SADABS*; Sheldrick, 1996)	*h* = −16→16
*T*_min_ = 0.79, *T*_max_ = 0.96	*k* = −27→26
32833 measured reflections	*l* = −10→10

### Refinement

**Table:**

Refinement on *F*^2^	Primary atom site location: structure-invariant direct methods
Least-squares matrix: full	Secondary atom site location: difference Fourier map
*R*[*F*^2^ > 2σ(*F*^2^)] = 0.040	Hydrogen site location: inferred from neighbouring sites
*wR*(*F*^2^) = 0.116	H atoms treated by a mixture of independent and constrained refinement
*S* = 1.06	*w* = 1/[σ^2^(*F*_o_^2^) + (0.0567*P*)^2^ + 0.8384*P*] where *P* = (*F*_o_^2^ + 2*F*_c_^2^)/3
4749 reflections	(Δ/σ)_max_ < 0.001
314 parameters	Δρ_max_ = 0.49 e Å^−^^3^
6 restraints	Δρ_min_ = −0.36 e Å^−^^3^

### Special details

**Table:**

Geometry. All esds (except the esd in the dihedral angle between two l.s. planes) are estimated using the full covariance matrix. The cell esds are taken into account individually in the estimation of esds in distances, angles and torsion angles; correlations between esds in cell parameters are only used when they are defined by crystal symmetry. An approximate (isotropic) treatment of cell esds is used for estimating esds involving l.s. planes.
Refinement. Refinement of F^2^ against ALL reflections. The weighted R-factor wR and goodness of fit S are based on F^2^, conventional R-factors R are based on F, with F set to zero for negative F^2^. The threshold expression of F^2^ > 2sigma(F^2^) is used only for calculating R-factors(gt) etc. and is not relevant to the choice of reflections for refinement. R-factors based on F^2^ are statistically about twice as large as those based on F, and R- factors based on ALL data will be even larger.

### Fractional atomic coordinates and isotropic or equivalent isotropic displacement parameters (Å^2^)

**Table:**

	*x*	*y*	*z*	*U*_iso_*/*U*_eq_	
C1	0.30225 (18)	0.17206 (10)	0.3731 (3)	0.0475 (5)	
C2	0.29999 (18)	0.23603 (10)	0.3655 (3)	0.0476 (5)	
H2	0.3516	0.2588	0.4226	0.057*	
C3	0.22127 (18)	0.26696 (10)	0.2732 (3)	0.0483 (5)	
C4	0.1448 (2)	0.23178 (13)	0.1905 (3)	0.0602 (6)	
H4	0.0917	0.2513	0.1278	0.072*	
C5	0.1469 (2)	0.16794 (13)	0.2007 (4)	0.0679 (7)	
H5	0.0942	0.1450	0.1465	0.081*	
C6	0.2257 (2)	0.13783 (11)	0.2896 (3)	0.0598 (6)	
H6	0.2274	0.0948	0.2933	0.072*	
C7	0.3846 (2)	0.08228 (10)	0.4912 (3)	0.0551 (6)	
H7A	0.3142	0.0692	0.5105	0.066*	
H7B	0.4282	0.0733	0.5885	0.066*	
C8	0.42315 (18)	0.04390 (11)	0.3518 (3)	0.0481 (5)	
C9	0.21649 (18)	0.33439 (11)	0.2639 (3)	0.0510 (5)	
H9	0.1681	0.3509	0.1872	0.061*	
C10	0.27286 (19)	0.37450 (11)	0.3519 (3)	0.0532 (6)	
H10	0.3202	0.3598	0.4327	0.064*	
C11	0.2635 (2)	0.44275 (11)	0.3265 (3)	0.0532 (6)	
C12	0.8386 (2)	0.06615 (14)	−0.0461 (4)	0.0743 (8)	
H12	0.8277	0.0236	−0.0436	0.089*	
C13	0.9260 (3)	0.08874 (17)	−0.1225 (4)	0.0839 (9)	
H13	0.9721	0.0617	−0.1683	0.101*	
C14	0.9410 (2)	0.15085 (16)	−0.1275 (4)	0.0753 (8)	
H14	0.9983	0.1667	−0.1771	0.090*	
C15	0.87155 (19)	0.19118 (13)	−0.0591 (3)	0.0584 (6)	
C16	0.78703 (17)	0.16457 (11)	0.0156 (3)	0.0488 (5)	
C17	0.8803 (2)	0.25730 (14)	−0.0638 (3)	0.0672 (7)	
H17	0.9364	0.2750	−0.1124	0.081*	
C18	0.8103 (2)	0.29410 (13)	−0.0002 (3)	0.0664 (7)	
H18	0.8184	0.3368	−0.0056	0.080*	
C19	0.72305 (19)	0.26889 (11)	0.0762 (3)	0.0531 (6)	
C20	0.71149 (17)	0.20406 (10)	0.0847 (3)	0.0452 (5)	
C21	0.6466 (2)	0.30519 (11)	0.1432 (3)	0.0627 (7)	
H21	0.6516	0.3481	0.1407	0.075*	
C22	0.5655 (2)	0.27787 (12)	0.2118 (3)	0.0638 (7)	
H22	0.5141	0.3018	0.2556	0.077*	
C23	0.5599 (2)	0.21306 (11)	0.2158 (3)	0.0545 (6)	
H23	0.5042	0.1947	0.2639	0.065*	
Mn1	0.62539 (3)	0.071726 (15)	0.14801 (4)	0.04754 (13)	
N1	0.77147 (16)	0.10253 (9)	0.0220 (2)	0.0554 (5)	
N2	0.63039 (14)	0.17695 (8)	0.1542 (2)	0.0465 (4)	
O1	0.38426 (13)	0.14746 (7)	0.46548 (19)	0.0546 (4)	
O2	0.48009 (14)	0.06992 (8)	0.2560 (2)	0.0619 (5)	
O3	0.39735 (14)	−0.01191 (7)	0.34978 (19)	0.0587 (4)	
O4	0.18729 (16)	0.46409 (9)	0.2449 (3)	0.0775 (6)	
O5	0.33793 (14)	0.47531 (7)	0.3879 (2)	0.0547 (4)	
O1W	0.70675 (16)	0.06432 (8)	0.3947 (2)	0.0615 (5)	
H1WA	0.674 (2)	0.0477 (14)	0.467 (3)	0.092*	
H1WB	0.7569 (19)	0.0409 (14)	0.374 (4)	0.092*	
O2W	0.53707 (14)	0.07366 (7)	−0.0852 (2)	0.0524 (4)	
H2WA	0.4737 (14)	0.0643 (13)	−0.080 (4)	0.079*	
H2WB	0.559 (2)	0.0562 (13)	−0.165 (3)	0.079*	
O3W	0.0161 (11)	0.5474 (5)	0.0875 (16)	0.494 (7)*	

### Atomic displacement parameters (Å^2^)

**Table:**

	*U*^11^	*U*^22^	*U*^33^	*U*^12^	*U*^13^	*U*^23^
C1	0.0543 (13)	0.0511 (12)	0.0388 (12)	0.0027 (10)	0.0148 (10)	−0.0019 (9)
C2	0.0522 (12)	0.0478 (12)	0.0440 (12)	−0.0016 (10)	0.0121 (10)	−0.0025 (9)
C3	0.0541 (13)	0.0526 (12)	0.0399 (12)	−0.0012 (10)	0.0161 (10)	0.0002 (9)
C4	0.0590 (15)	0.0682 (16)	0.0530 (15)	0.0047 (12)	0.0017 (12)	−0.0013 (12)
C5	0.0645 (16)	0.0671 (16)	0.0714 (18)	−0.0061 (13)	−0.0019 (14)	−0.0165 (14)
C6	0.0670 (16)	0.0503 (13)	0.0625 (16)	−0.0018 (11)	0.0075 (13)	−0.0069 (11)
C7	0.0758 (16)	0.0466 (12)	0.0440 (13)	0.0073 (11)	0.0115 (11)	0.0021 (10)
C8	0.0537 (13)	0.0518 (13)	0.0393 (12)	0.0036 (10)	0.0070 (10)	0.0041 (10)
C9	0.0521 (13)	0.0529 (13)	0.0496 (13)	0.0042 (10)	0.0146 (10)	0.0077 (10)
C10	0.0610 (14)	0.0496 (12)	0.0502 (14)	0.0030 (11)	0.0132 (11)	0.0078 (10)
C11	0.0592 (14)	0.0512 (13)	0.0509 (14)	0.0067 (11)	0.0157 (11)	0.0056 (11)
C12	0.0819 (19)	0.0678 (17)	0.076 (2)	0.0031 (14)	0.0293 (16)	0.0045 (14)
C13	0.078 (2)	0.098 (2)	0.080 (2)	0.0136 (18)	0.0354 (17)	0.0091 (18)
C14	0.0615 (17)	0.094 (2)	0.0725 (19)	−0.0057 (15)	0.0185 (14)	0.0217 (16)
C15	0.0479 (13)	0.0779 (17)	0.0488 (14)	−0.0080 (12)	−0.0006 (11)	0.0162 (12)
C16	0.0492 (12)	0.0584 (13)	0.0380 (12)	−0.0090 (10)	−0.0028 (10)	0.0082 (10)
C17	0.0588 (15)	0.0775 (18)	0.0646 (17)	−0.0234 (14)	−0.0025 (13)	0.0241 (14)
C18	0.0718 (17)	0.0603 (15)	0.0657 (17)	−0.0182 (14)	−0.0071 (14)	0.0180 (13)
C19	0.0627 (14)	0.0505 (12)	0.0443 (13)	−0.0125 (11)	−0.0107 (11)	0.0095 (10)
C20	0.0491 (12)	0.0507 (12)	0.0348 (11)	−0.0075 (10)	−0.0055 (9)	0.0061 (9)
C21	0.0886 (19)	0.0417 (12)	0.0569 (16)	−0.0027 (12)	−0.0032 (14)	0.0038 (11)
C22	0.0814 (18)	0.0524 (14)	0.0577 (16)	0.0066 (13)	0.0061 (14)	0.0009 (12)
C23	0.0629 (15)	0.0512 (13)	0.0504 (14)	−0.0013 (11)	0.0104 (11)	−0.0004 (10)
Mn1	0.0605 (2)	0.0416 (2)	0.0417 (2)	−0.00718 (15)	0.01285 (15)	−0.00287 (14)
N1	0.0597 (12)	0.0562 (12)	0.0518 (12)	−0.0008 (9)	0.0161 (9)	0.0028 (9)
N2	0.0546 (11)	0.0461 (10)	0.0387 (10)	−0.0064 (8)	0.0030 (8)	0.0005 (8)
O1	0.0657 (10)	0.0470 (8)	0.0511 (9)	0.0031 (7)	0.0034 (8)	−0.0002 (7)
O2	0.0706 (11)	0.0645 (11)	0.0533 (10)	−0.0061 (8)	0.0242 (9)	0.0011 (8)
O3	0.0816 (12)	0.0501 (9)	0.0460 (9)	−0.0035 (8)	0.0173 (8)	−0.0021 (7)
O4	0.0698 (12)	0.0597 (11)	0.1015 (16)	0.0120 (9)	−0.0055 (11)	0.0033 (11)
O5	0.0661 (10)	0.0442 (8)	0.0543 (10)	0.0009 (8)	0.0075 (8)	0.0054 (7)
O1W	0.0820 (13)	0.0525 (10)	0.0502 (10)	−0.0099 (8)	0.0055 (9)	−0.0012 (8)
O2W	0.0640 (10)	0.0531 (9)	0.0410 (9)	−0.0064 (8)	0.0106 (8)	−0.0053 (7)

### Geometric parameters (Å, º)

**Table:**

C1—O1	1.368 (3)	C14—H14	0.9300
C1—C6	1.379 (3)	C15—C16	1.406 (3)
C1—C2	1.383 (3)	C15—C17	1.433 (4)
C2—C3	1.397 (3)	C16—N1	1.356 (3)
C2—H2	0.9300	C16—C20	1.438 (3)
C3—C4	1.386 (3)	C17—C18	1.333 (4)
C3—C9	1.459 (3)	C17—H17	0.9300
C4—C5	1.381 (4)	C18—C19	1.429 (4)
C4—H4	0.9300	C18—H18	0.9300
C5—C6	1.375 (4)	C19—C21	1.398 (4)
C5—H5	0.9300	C19—C20	1.410 (3)
C6—H6	0.9300	C20—N2	1.355 (3)
C7—O1	1.423 (3)	C21—C22	1.354 (4)
C7—C8	1.528 (3)	C21—H21	0.9300
C7—H7A	0.9700	C22—C23	1.402 (3)
C7—H7B	0.9700	C22—H22	0.9300
C8—O2	1.247 (3)	C23—N2	1.322 (3)
C8—O3	1.250 (3)	C23—H23	0.9300
C9—C10	1.317 (3)	Mn1—O2	2.1209 (17)
C9—H9	0.9300	Mn1—O5^i^	2.1594 (16)
C10—C11	1.493 (3)	Mn1—O2W	2.1731 (17)
C10—H10	0.9300	Mn1—O1W	2.2364 (19)
C11—O4	1.241 (3)	Mn1—N2	2.2738 (18)
C11—O5	1.267 (3)	Mn1—N1	2.303 (2)
C12—N1	1.319 (3)	O5—Mn1^ii^	2.1594 (16)
C12—C13	1.410 (4)	O1W—H1WA	0.835 (17)
C12—H12	0.9300	O1W—H1WB	0.845 (17)
C13—C14	1.356 (4)	O2W—H2WA	0.842 (16)
C13—H13	0.9300	O2W—H2WB	0.828 (16)
C14—C15	1.393 (4)		
			
O1—C1—C6	124.7 (2)	C18—C17—C15	121.8 (2)
O1—C1—C2	115.3 (2)	C18—C17—H17	119.1
C6—C1—C2	120.0 (2)	C15—C17—H17	119.1
C1—C2—C3	121.0 (2)	C17—C18—C19	121.0 (2)
C1—C2—H2	119.5	C17—C18—H18	119.5
C3—C2—H2	119.5	C19—C18—H18	119.5
C4—C3—C2	118.2 (2)	C21—C19—C20	117.3 (2)
C4—C3—C9	119.7 (2)	C21—C19—C18	123.5 (2)
C2—C3—C9	122.1 (2)	C20—C19—C18	119.2 (2)
C5—C4—C3	120.4 (2)	N2—C20—C19	122.4 (2)
C5—C4—H4	119.8	N2—C20—C16	118.01 (19)
C3—C4—H4	119.8	C19—C20—C16	119.5 (2)
C6—C5—C4	121.0 (2)	C22—C21—C19	120.1 (2)
C6—C5—H5	119.5	C22—C21—H21	120.0
C4—C5—H5	119.5	C19—C21—H21	120.0
C5—C6—C1	119.4 (2)	C21—C22—C23	119.1 (3)
C5—C6—H6	120.3	C21—C22—H22	120.4
C1—C6—H6	120.3	C23—C22—H22	120.4
O1—C7—C8	114.93 (19)	N2—C23—C22	122.9 (2)
O1—C7—H7A	108.5	N2—C23—H23	118.6
C8—C7—H7A	108.5	C22—C23—H23	118.6
O1—C7—H7B	108.5	O2—Mn1—O5^i^	104.24 (7)
C8—C7—H7B	108.5	O2—Mn1—O2W	87.18 (7)
H7A—C7—H7B	107.5	O5^i^—Mn1—O2W	90.14 (6)
O2—C8—O3	126.3 (2)	O2—Mn1—O1W	89.24 (7)
O2—C8—C7	117.9 (2)	O5^i^—Mn1—O1W	87.90 (6)
O3—C8—C7	115.8 (2)	O2W—Mn1—O1W	175.37 (7)
C10—C9—C3	127.4 (2)	O2—Mn1—N2	91.90 (7)
C10—C9—H9	116.3	O5^i^—Mn1—N2	163.86 (7)
C3—C9—H9	116.3	O2W—Mn1—N2	90.76 (6)
C9—C10—C11	122.4 (2)	O1W—Mn1—N2	92.29 (6)
C9—C10—H10	118.8	O2—Mn1—N1	163.93 (7)
C11—C10—H10	118.8	O5^i^—Mn1—N1	91.42 (7)
O4—C11—O5	124.1 (2)	O2W—Mn1—N1	89.25 (7)
O4—C11—C10	119.8 (2)	O1W—Mn1—N1	94.99 (7)
O5—C11—C10	116.1 (2)	N2—Mn1—N1	72.48 (7)
N1—C12—C13	123.1 (3)	C12—N1—C16	118.0 (2)
N1—C12—H12	118.4	C12—N1—Mn1	126.53 (18)
C13—C12—H12	118.4	C16—N1—Mn1	115.43 (15)
C14—C13—C12	118.4 (3)	C23—N2—C20	118.3 (2)
C14—C13—H13	120.8	C23—N2—Mn1	125.40 (16)
C12—C13—H13	120.8	C20—N2—Mn1	116.33 (15)
C13—C14—C15	120.6 (3)	C1—O1—C7	117.52 (19)
C13—C14—H14	119.7	C8—O2—Mn1	147.61 (17)
C15—C14—H14	119.7	C11—O5—Mn1^ii^	130.24 (15)
C14—C15—C16	117.1 (3)	Mn1—O1W—H1WA	117 (2)
C14—C15—C17	124.0 (2)	Mn1—O1W—H1WB	100 (2)
C16—C15—C17	118.9 (3)	H1WA—O1W—H1WB	108 (2)
N1—C16—C15	122.7 (2)	Mn1—O2W—H2WA	114 (2)
N1—C16—C20	117.7 (2)	Mn1—O2W—H2WB	121 (2)
C15—C16—C20	119.5 (2)	H2WA—O2W—H2WB	108 (2)
			
O1—C1—C2—C3	178.98 (19)	C13—C12—N1—Mn1	179.4 (2)
C6—C1—C2—C3	−0.4 (3)	C15—C16—N1—C12	−0.5 (4)
C1—C2—C3—C4	0.6 (3)	C20—C16—N1—C12	177.7 (2)
C1—C2—C3—C9	179.5 (2)	C15—C16—N1—Mn1	−179.03 (17)
C2—C3—C4—C5	0.3 (4)	C20—C16—N1—Mn1	−0.8 (2)
C9—C3—C4—C5	−178.6 (2)	O2—Mn1—N1—C12	−164.1 (3)
C3—C4—C5—C6	−1.4 (4)	O5^i^—Mn1—N1—C12	3.1 (2)
C4—C5—C6—C1	1.7 (4)	O2W—Mn1—N1—C12	−87.0 (2)
O1—C1—C6—C5	180.0 (2)	O1W—Mn1—N1—C12	91.1 (2)
C2—C1—C6—C5	−0.8 (4)	N2—Mn1—N1—C12	−178.1 (3)
O1—C7—C8—O2	22.3 (3)	O2—Mn1—N1—C16	14.3 (4)
O1—C7—C8—O3	−160.6 (2)	O5^i^—Mn1—N1—C16	−178.49 (16)
C4—C3—C9—C10	169.4 (2)	O2W—Mn1—N1—C16	91.39 (16)
C2—C3—C9—C10	−9.5 (4)	O1W—Mn1—N1—C16	−90.47 (16)
C3—C9—C10—C11	177.6 (2)	N2—Mn1—N1—C16	0.36 (15)
C9—C10—C11—O4	15.0 (4)	C22—C23—N2—C20	0.2 (3)
C9—C10—C11—O5	−162.8 (2)	C22—C23—N2—Mn1	−177.99 (18)
N1—C12—C13—C14	−0.7 (5)	C19—C20—N2—C23	0.1 (3)
C12—C13—C14—C15	−0.2 (5)	C16—C20—N2—C23	−179.0 (2)
C13—C14—C15—C16	0.6 (4)	C19—C20—N2—Mn1	178.38 (15)
C13—C14—C15—C17	−178.0 (3)	C16—C20—N2—Mn1	−0.7 (2)
C14—C15—C16—N1	−0.3 (3)	O2—Mn1—N2—C23	2.19 (19)
C17—C15—C16—N1	178.4 (2)	O5^i^—Mn1—N2—C23	−177.5 (2)
C14—C15—C16—C20	−178.5 (2)	O2W—Mn1—N2—C23	89.39 (18)
C17—C15—C16—C20	0.2 (3)	O1W—Mn1—N2—C23	−87.13 (19)
C14—C15—C17—C18	178.2 (3)	N1—Mn1—N2—C23	178.4 (2)
C16—C15—C17—C18	−0.4 (4)	O2—Mn1—N2—C20	−175.99 (15)
C15—C17—C18—C19	0.2 (4)	O5^i^—Mn1—N2—C20	4.3 (3)
C17—C18—C19—C21	−179.2 (2)	O2W—Mn1—N2—C20	−88.79 (15)
C17—C18—C19—C20	0.3 (4)	O1W—Mn1—N2—C20	94.69 (15)
C21—C19—C20—N2	0.1 (3)	N1—Mn1—N2—C20	0.18 (14)
C18—C19—C20—N2	−179.5 (2)	C6—C1—O1—C7	−7.0 (3)
C21—C19—C20—C16	179.1 (2)	C2—C1—O1—C7	173.64 (19)
C18—C19—C20—C16	−0.5 (3)	C8—C7—O1—C1	80.7 (3)
N1—C16—C20—N2	1.0 (3)	O3—C8—O2—Mn1	−54.9 (4)
C15—C16—C20—N2	179.28 (19)	C7—C8—O2—Mn1	121.9 (3)
N1—C16—C20—C19	−178.06 (19)	O5^i^—Mn1—O2—C8	37.3 (3)
C15—C16—C20—C19	0.2 (3)	O2W—Mn1—O2—C8	126.8 (3)
C20—C19—C21—C22	−0.4 (3)	O1W—Mn1—O2—C8	−50.3 (3)
C18—C19—C21—C22	179.1 (2)	N2—Mn1—O2—C8	−142.6 (3)
C19—C21—C22—C23	0.6 (4)	N1—Mn1—O2—C8	−155.9 (3)
C21—C22—C23—N2	−0.5 (4)	O4—C11—O5—Mn1^ii^	−9.6 (4)
C13—C12—N1—C16	1.0 (4)	C10—C11—O5—Mn1^ii^	168.14 (15)

Symmetry codes: (i) −*x*+1, *y*−1/2, −*z*+1/2; (ii) −*x*+1, *y*+1/2, −*z*+1/2.

### Hydrogen-bond geometry (Å, º)

**Table:**

*D*—H···*A*	*D*—H	H···*A*	*D*···*A*	*D*—H···*A*
O2*W*—H2*WB*···O3^iii^	0.83 (2)	1.92 (2)	2.741 (2)	176 (3)
O2*W*—H2*WA*···O5^iv^	0.84 (2)	1.95 (2)	2.764 (2)	164 (3)
O1*W*—H1*WB*···O4^i^	0.85 (2)	2.08 (2)	2.841 (3)	150 (3)
O1*W*—H1*WA*···O3^v^	0.84 (2)	1.98 (2)	2.810 (2)	176 (3)

Symmetry codes: (i) −*x*+1, *y*−1/2, −*z*+1/2; (iii) −*x*+1, −*y*, −*z*; (iv) *x*, −*y*+1/2, *z*−1/2; (v) −*x*+1, −*y*, −*z*+1.
